# Effects of *Rhodomyrtus tomentosa* Extract on Killing Activity of Human Neutrophils and Membrane Integrity of Enterohaemorrhagic *Escherichia coli* O157:H7

**DOI:** 10.3390/molecules21060692

**Published:** 2016-05-27

**Authors:** Jutharat Hmoteh, Khadar Syed Musthafa, Rattanaruji Pomwised, Supayang Piyawan Voravuthikunchai

**Affiliations:** 1 Department of Microbiology, Faculty of Science, Prince of Songkla University, Hat Yai, Songkhla 90112, Thailand; bebegamalar@gmail.com (J.H.); rattanaruji.p@psu.ac.th (R.P.); 2 Excellent Research Laboratory on Natural products, Faculty of Science and Natural Product Research Center of Excellence, Prince of Songkla University, Hat Yai, Songkhla 90112, Thailand; syed_musthafa@rediffmail.com

**Keywords:** *Rhodomyrtus tomentosa*, human neutrophils, *Escherichia coli* O157:H7, immunomodulatory activity, membrane integrity

## Abstract

Enterohaemorrhagic *Escherichia coli* (*E. coli*) O157:H7 is one of the most virulent causative agents of foodborne disease. Use of antibiotics for the treatment against *E. coli* O157:H7 infection leads to hemolytic uremic syndrome. The present study evaluated the potential of ethanolic leaf extract of a medicinal plant, *Rhodomyrtus tomentosa* in enhancing the killing activity of human neutrophils against *E. coli* O157:H7. In addition, the effects of the extract on membrane permeability of the organisms were studied. In the killing assay, percentage survival of the bacterial cells after being exposed to human neutrophils in the presence of various concentrations of the extract were determined. At 45 min, percentage survival of *E. coli* O157:H7 and *E. coli* ATCC 25922 after treated with neutrophils in the presence of the extract at 125–250 µg/mL was 58.48%–50.28% and 69.13%–35.35%, respectively. Furthermore, upon treatment with *R.* *tomentosa* at 250 µg/mL uptake of crystal violet by *E. coli* O157:H7 and *E. coli* ATCC 25922 was increased to 40.07% and 36.16%, respectively. Therefore, it is suggested that the extract exhibited dual effects as immunostimulant and membrane permeabilizing agent perhaps resulted in enhancing the killing activity of neutrophils against the organisms.

## 1. Introduction

Enterohaemorrhagic *Escherichia coli* O157:H7 is a Gram-negative human pathogen that causes bloody diarrhea as well leads to severe abdominal cramps in the infected patients. Moreover, production of Shiga toxin could lead to hemorrhagic colitis in humans [1,2]. Although conventional antimicrobial treatment is generally prescribed for the infection caused by various pathogenic bacteria, use of antibiotics to treat enterohaemorrhagic *E. coli* O157:H7 infection is not generally recommended due to an increased risk of hemolytic uremic syndrome [3]. Therefore, suitable alternative measures for the prevention of infections caused by *E. coli* O157:H7 are urgently required [4].

Plants are valuable sources of pharmacologically important bioactive metabolites and, therefore, targeting the resource is one among several interesting alternative strategies to control enterohaemorrhagic *E. coli* O157:H7 infection [5,6]. Utilization of plant materials as an immunostimulant to increase the activity of the host immune system against a pathogenic organism is an interesting approach to eliminate microbial infection within the host system. For example, a polysaccharide fraction from *Solanum nigrum* Linn [7] and *Coffea arabica* L. seed extract [8] was reported previously to activate the host immune response. In addition, it has been well demonstrated that the bacterial cell membrane is considered as a defensive barrier known to protect the pathogenic bacteria from environmental stress, and, therefore, plant extracts possessing an ability to alter bacterial membrane integrity is another strategy to make the pathogen more susceptible to the killing activity of host immune cells or antimicrobials (plant derived drugs) [9,10]. In view of this point, plants exhibiting immunostimulatory effects or have an ability to alter membrane integrity could be exploited to eliminate *E. coli* O157:H7 infections.

*Rhodomyrtus tomentosa* (Aiton) Hassk. is a medicinal plant belonging to the Myrtaceae family. The plant has been utilized for the remedy of various infectious diseases including urinary tract infection [11], diarrhea [12], and dysmenorrhea [13]. Furthermore, leaf extract of the plant demonstrated various biological activities including antioxidant [14,15], antibacterial [16,17], and antibiofilm properties [18]. Therefore, considering the potential of *R.* *tomentosa* extract, in the present study, an attempt was made to validate the effect of the extract in enhancing the killing activity of human neutrophils against *E. coli* O157:H7. Besides, effects of the extract on membrane integrity of the pathogen were evaluated.

## 2. Results and Discussion

### 2.1. Effects of R. tomentosa Extract on Killing Activity of Neutrophils

**Figure 1:**
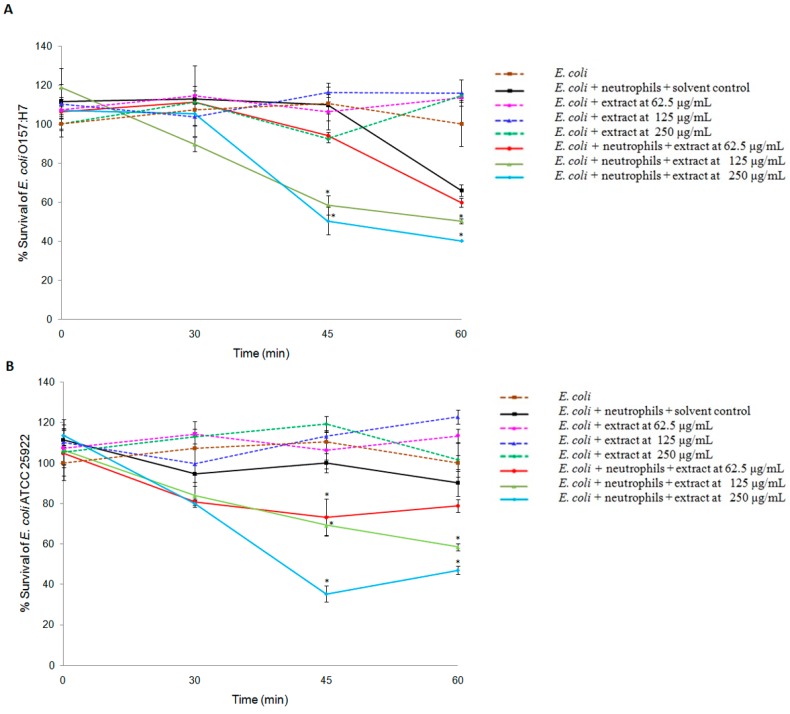
Effects of *R.* *tomentosa* extract on the killing activity of human neutrophils against *Escherichia coli* O157:H7 RIMD 05091078 (**A**) and *E. coli* ATCC 25922 (**B**). The bacterial cells were exposed to neutrophils in the absence and presence of *R.* *tomentosa* extract (62.5–250 µg/mL). *Escherichia coli* cells in the presence of the extract (62.5–250 µg/mL) alone were also maintained. Dimethyl sulfoxide (0.5% *v*/*v*) was used as solvent control. The percentage survival of the organisms was determined at 0, 30, 45, and 60 min. Data are expressed as mean ± standard error of the mean from two independent experiments. Each measure was performed in triplicate. * Statistical significance at *p* < 0.05.

Immunostimulatory activity of plants in enhancing the killing effect of the host immune cells is an attractive approach to overcome the usage of antibiotics in controlling the infection caused by pathogenic bacteria [9,10]. Therefore, in the present study, potential of the ethanolic leaf extract of *R.* *tomentosa* was evaluated for its ability in increasing killing activity of human neutrophils against *E. coli* O157:H7. The percentage survival of *E. coli* after exposed to neutrophils in the presence of the extract is given in Figure 1A and B. It was observed that the extract alone at the tested concentrations of 62.5–250 μg/mL had no direct killing activity on the organisms. However, the extract enhanced the killing activity of neutrophils towards *E. coli*. At 30 min, the extract at 62.5–250 µg/mL showed a moderate effect in enhancing the killing activity of neutrophils against both *E. coli* strains. However, a significant increase in the activity of neutrophils was observed after 45 and 60 min (*p* < 0.05). At 45 min, survival percentage of *E. coli* O157:H7 and *E. coli* ATCC 25922 when exposed to neutrophils in the presence of 62.5, 125, and 250 µg/mL of the extract were 94.15%, 58.48%, 50.28%, and 73.15%, 69.13%, and 35.35 %, respectively. Similarly, at 60 min, survival percentage of *E. coli* O157:H7 and *E. coli* ATCC 25922 after being incubated with neutrophils in the presence of the same concentrations of the extract was 59.91%, 50.34%, 40.15% and 78.79%, 58.46%, and 47.02%, respectively. In the earlier study, it was demonstrated that DMSO up to 1% did not affect the killing activity of human neutrophils against *E. coli* [19]. In the present work, the solvent was used at the highest concentration of 0.5% (*v*/*v*) for control and treatment samples. From the results of cell survival assay, the killing activity of neutrophils increased with the increased addition of the extract. Therefore, it is envisaged that the solvent at the tested percentage did not interfere with the observed effects with neutrophils and demonstrated the ability of *R. tomentosa* extract to enhance the killing activity of human neutrophils against *E. coli*. In the earlier studies, other plant resources have been reported to enhance the activity of host immune cells against pathogenic organisms. Serafino *et al.* [20] reported that, upon treatment with *Eucalyptus* essential oil, the phagocytic ability of macrophages increased towards *Staphylococcus aureus*. In another study, iridoids fraction obtained from methanol extract of *Barleria prionitis* enhanced the intracellular killing activity of neutrophils against *Candida albicans* [10].

### 2.2. Effects of R. tomentosa Extract on Membrane Integrity of E. coli

**Figure 2:**
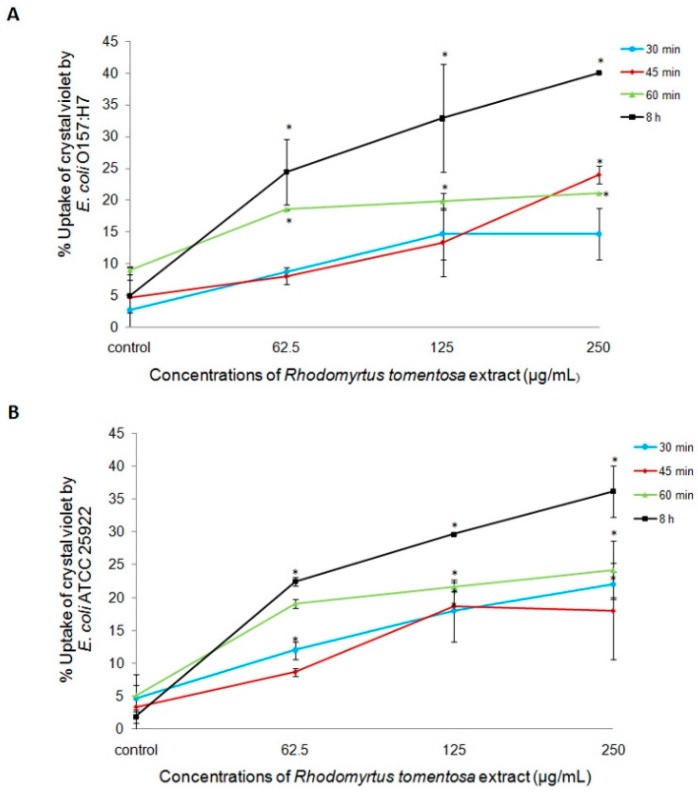
Effects of *R.* *tomentosa* extract on membrane permeability of *Escherichia coli* O157:H7 RIMD 05091078 (**A**) and *E. coli* ATCC 25922 (**B**) Crystal violet uptake by *E. coli* at 30, 45, 60 min, and 8 h after treated with the extract at 62.5, 125, and 250 µg/mL was determined. Dimethyl sulfoxide (0.5% *v*/*v*) was used as the solvent control. Data are expressed as mean ± standard error from two independent experiments. Each measure was performed in triplicate. * Statistical significance at *p* < 0.05.

Membrane permeability or integrity plays a critical role as a barrier in Gram-negative bacteria which protects the bacterial cells from the action of antimicrobial compounds [9,10]. Alteration in the membrane permeability could lead to easy passage of antimicrobial drugs into the cells resulting cell death [9,10,21,22]. In addition, defects in the membrane integrity may increase the susceptibility of the bacterial cells to host immune attack. In the present study, since the extract alone at the tested concentrations did not produce any considerable effects on the survival of *E. coli* cells, we evaluated the possible impact of *R. tomentosa* extract against the organisms by membrane permeability assay at the time intervals as those used in a similar set up mentioned in cell survival assay. It was observed that, upon treatment with the extract, uptake of crystal violet by the cell membrane of the organisms was significantly increased. Crystal violet uptake by *E. coli* O157:H7 and *E. coli* ATCC 25922 after 8 h was 4.89% and 1.95%, respectively. However, upon being treated with the extract at 62.5–250 µg/mL, uptake of the dye was 24.43%–40.07% in *E. coli* O157:H7 and 22.48%–36.16% in *E. coli* ATCC 25922. Even after short time exposure to *R. tomentosa* extract, the organisms showed alteration in their membrane permeability. The uptake of dye by *E. coli* O157:H7 at the similar time interval mentioned in the cell survival assay at 30, 45, and 60 min after the extract treatment at 62.5–250 µg/mL was 8.67%–14.67%, 8.00%–24.00%, and 18.47%–21.02%, respectively. Similarly, the uptake of dye by *E. coli* ATCC 25922 at 30, 45, and 60 min after the extract treatment at 62.5–250 µg/mL was 12.00%–22.00%, 8.67%–18.00%, and 19.11%–24.20%, respectively (Figure 2A,B).

Therefore, it is demonstrated that the extract exhibited membrane permeabilizing activity rather than antibacterial activity at the tested concentrations, and resulted in the alteration of membrane integrity of *E. coli*. The attained result is in accordance with the previous study, in which membrane permeability of *E. coli* was disrupted after being treated with corilagin; a tannin group of compound resulted in increased uptake of crystal violet dye [22]. In another report, eugenol, a phytochemical from clove essential oil affected the membrane integrity of *S. aureus* [21]. We would also like to mention here that in our previous report, an acylphloroglucinol component, rhodomyrtone from the leaf extract of *R. tomentosa* showed immunomodulatory effects on THP-1 human monocyte cell line leading to enhanced killing of methicillin-resistant *Staphylococcus aureus* [23]. Therefore, it is anticipated that the extract possesses both membrane permeabilizing as well as immunomodulatory properties resulting in the increased susceptibility of *E. coli* cells to immune cell attack.

## 3. Experimental Section

### 3.1. Pathogens Used and Culture Conditions

Enterohaemorrhagic *Escherichia coli* O157:H7 RIMD 05091078 and *E. coli* ATCC 25922 were used as target strains. The strains were cultured in tryptic soy broth (TSB, Difco, Detroit, MI, USA) at 37 °C for 18 h. After incubation, the cells were pelleted at 2000× *g* for 7 min, washed twice with phosphate buffer saline (PBS) (pH 7.4). The cell pellet was suspended in Roswell Park Memorial Institute 1640 medium (RPMI-1640, Sigma-Aldrich, St. Louis, MO, USA) and used for opsonization.

### 3.2. Preparation of R. tomentosa Leaf Extract

*Rhodomyrtus tomentosa* leaves were collected in April, 2013 from Sadao District, Songkhla Province in the southern part of Thailand. The collected samples were dried, ground into a powder, and then soaked with 95% ethanol for seven days. The ethanol layer was collected and evaporated to complete dryness under a rotary evaporator. The dried ethanolic extract was dissolved in 100% dimethyl sulfoxide (DMSO, Merck, Darmstadt, Germany).

### 3.3. Serum Preparation and Opsonization

Fresh serum was obtained from normal AB blood group donors. The bacterial cell suspension in RPMI-1640 medium was incubated with 10% AB serum for 30 min at 37 °C. After incubation, the cell suspension was centrifuged at 2000× *g* for 10 min and washed twice with PBS. The cell density was adjusted to 10^8^ cfu/mL using RPMI 1640 medium. The numbers of opsonized cells were counted using a haemocytometer.

### 3.4. Isolation of Human Neutrophils

The buffy coat of healthy volunteers was obtained from the Blood bank of Songklanagarind hospital, and added with 6% dextran (Fluka, Buchs, Switzerland). The mixture was gently shaken and allowed to stand at room temperature for 1 h to sediment erythrocytes. Leukocytes from the upper layer were transferred to a sterile centrifuge tube. The protocol of Histopaque-1.077 (Sigma-Aldrich) was used with slight modification for the isolation of neutrophils. Briefly, leukocytes were layered in Histopaque-1.077 solution and centrifuged at 700× *g* for 35 min at 20 °C to isolate neutophils. After the recovery, the viability and purity of the neutrophils were determined by trypan blue (Difco) exclusion assay [24] and Wright Giemsa stain (Sigma-Aldrich), respectively. The viability and purity of the neutrophils were ≥98% and ≥95%, respectively.

### 3.5. Cell Survival Assay

The assay was performed by following the previously described method [10] with slight modification. In brief, *R. tomentosa* extract (stock solution—50 mg extract/mL DMSO) was double diluted in 1.5 mL eppendorf tubes containing 100 µL RPMI medium to yield concentrations ranging from 250 to 62.5 μg/mL. One hundred µL of neutrophils (10^6^ cells/mL) were added to each tube and finally 100 µL of the opsonized suspension of *E. coli* cells were added. The tube containing only neutrophils and *E. coli* along with the respective solvent was maintained as the control. In both control and treatment set up, the highest concentration of DMSO used was 0.5% (*v*/*v*). The tubes were incubated at 37 °C for 30, 45, 60 min with gentle shaking to expose *E. coli* to the extract and neutrophils. After incubation, 10 µL of the treated and untreated *E. coli* cell suspension was plated on tryptic soy agar and the colony forming units per milliliter (cfu/mL) was determined after incubation at 37 °C, overnight.

### 3.6. Membrane Permeability Assay

Effects of *R.* *tomentosa* extract on the integrity of *E. coli* were determined by membrane permeabilizing assay using crystal violet stain. The pathogen (10^8^ cells/mL) was incubated with the extract at 62.5, 125, and 250 µg/mL for 30, 45, and 60 min, and 8 h at 37 °C, and centrifuged at 9300× *g* for 5 min. The cell pellet was diluted with 0.5% sodium chloride (500 mg in 100 mL sterile water) solution containing 10 µg/mL of crystal violet. The content was incubated at 37 °C for 10 min, shaken and centrifuged at 13,400× *g* for 15 min. The absorbance of crystal violet in each supernatant was measured spectrophotometrically at OD_590_ [22].

### 3.7. Statistical Analysis

Statistical significance was calculated by analysis of variance (Anova). Comparisons between means were carried out according to the Dunnett test. *p* value < 0.05 was considered as significant difference.

## 4. Conclusions

In correlating the observed effects of *R.* *tomentosa* extract, it is believed that the extract causing alteration in the bacterial membrane integrity along with immunostimulatory effects probably enhanced the killing activity of neutrophils against *E. coli*. Further studies in exploring exact mechanism involving effects of *R.* *tomentosa* extract on enterohaemorrhagic *E. coli* O157:H7 could be useful in the treatment of the organism.
